# AI-Based Treatment Recommendations Enhance Speed and Accuracy in Bacteremia Management: A Comparative Study of Molecular and Phenotypic Data

**DOI:** 10.3390/life15060864

**Published:** 2025-05-27

**Authors:** Juan C. Gomez de la Torre, Ari Frenkel, Carlos Chavez-Lencinas, Alicia Rendon, José Alonso Cáceres, Luis Alvarado, Miguel Hueda-Zavaleta

**Affiliations:** 1 Clinical Laboratory Roe, Lima 15076, Peru; jcaceres@labroe.com (J.A.C.); lalvarado@labroe.com (L.A.); 2 Arkstone Medical Solutions, Boca Raton, FL 33428, USA; afrenkel@arkstonemedical.com (A.F.); arendon@arkstonemedical.com (A.R.); mighueda@virtual.upt.pe (M.H.-Z.); 3 Hospital Nacional Edgardo Rebagliati Martins, Lima 15073, Peru; cchavezl1@unmsm.edu.pe; 4 Department of Internal Medicine, San Fernando School of Medicine, Cathedral of Infectious Diseases, National University of San Marcos, Lima 15072, Peru; 5 Diagnóstico, Tratamiento e Investigación de Enfermedades Infecciosas y Tropicales, Universidad Privada de Tacna, Tacna 23003, Peru

**Keywords:** bacteremia, bloodstream infection, antimicrobial stewardship, artificial intelligence, clinical decision support systems, machine learning, molecular diagnostic, rapid diagnostic testing

## Abstract

Background: Bloodstream infections continue to pose a serious global health threat due to their high morbidity and mortality, further worsened by rising antimicrobial resistance and delays in starting targeted therapy. This study assesses the accuracy and timeliness of therapeutic recommendations produced by an artificial intelligence (AI)-driven and machine-learning (ML) clinical decision support system (CDSS), comparing results based on molecular diagnostics alone with those that combine molecular and phenotypic data (standard cultures). Methods: In a prospective cross-sectional study conducted in Lima, Peru, 117 blood cultures were analyzed using FilmArray/GeneXpert for molecular identification and MALDI-TOF/VITEK 2.0 for phenotypic profiling. The AI/ML-based CDSS provided treatment recommendations in two formats, which were assessed for concordance and turnaround time. Results: Therapeutic recommendations showed 80.3% consistency between data types, with 86.3% concordance in pathogen and resistance detection. Notably, molecular-only recommendations were delivered 29 h earlier than those incorporating phenotypic data. Escherichia coli was the most frequently isolated pathogen, with a 95% concordance in suggested therapy. A substantial agreement was observed in treatment consistency (Kappa = 0.80). Conclusions: These findings highlight the potential of using AI-powered CDSS in conjunction with molecular diagnostics to accelerate clinical decision-making in bacteremia, supporting more timely interventions and improved antimicrobial stewardship. Further research is warranted to assess scalability and impact across diverse clinical settings.

## 1. Introduction

Bloodstream infections (BSIs) represent a significant global health challenge, contributing substantially to morbidity, mortality, and increased healthcare costs [1,2]. The incidence of BSIs is often linked to conditions such as sepsis, septic shock, and multi-organ failure, leading to approximately 11 million deaths annually worldwide, with a disproportionate burden in resource-limited settings [3,4]. In locations such as Peru, the escalating prevalence of antimicrobial resistance (AMR) further exacerbates the challenges of managing BSIs, rendering treatment more complex and significantly increasing mortality rates [5].

Timely and appropriate antimicrobial therapy is crucial for managing BSIs, as delays can increase mortality rates [6,7]. Traditional diagnostic methods, relying on blood cultures and phenotypic susceptibility testing, typically require 48–72 h for pathogen identification and susceptibility determination [8,9]. This delay often hinders the ability to initiate early, targeted therapy, leading to poorer patient outcomes. Recent advances in diagnostic technologies offer promising solutions to this problem, with rapid molecular diagnostic platforms, such as FilmArray (BioFire Diagnostics, LLC, Salt Lake City, UT, USA) and GeneXpert (Cepheid, Sunnyvale, CA, USA), significantly reducing the time needed for pathogen identification and clinically relevant resistance gene detection compared to traditional methods [10,11,12,13].

Artificial intelligence (AI) and machine learning (ML) are now being integrated into clinical decision support systems (CDSS) to address limitations in solely molecular-based diagnostics and traditional methods [14,15,16]. AI’s ability to analyze complex datasets and provide actionable insights has been demonstrated in various infectious disease contexts [17,18,19,20]. However, the comparative performance of CDSS based solely on molecular data versus those incorporating integrated molecular and phenotypic data remains underexamined due to limited studies. While systems like Arkstone’s OneChoice Molecular report (AOCHMR), which generates therapeutic recommendations based on molecular diagnostic data, have shown promise, the extent to which they align with gold standards has not been evaluated [21,22].

This study addresses the knowledge gap by evaluating the precision of therapeutic recommendations generated by the AOCHMR compared to Arkstone’s OneChoice Fusion report (AOCHFR), which combines molecular and phenotypic data. Specifically, we seek to quantify the diagnostic accuracy and therapeutic concordance between these two approaches, within a cohort of patients with bacteremia in Lima, Peru. This will involve a prospective, cross-sectional analysis of 117 patients with bacteremia, where pathogen identification and antimicrobial susceptibility were determined using FilmArray/GeneXpert and MALDI-TOF/VITEK 2.0, respectively. AI and ML technologies were used to analyze the molecular data, including a validated machine learning algorithm. The study was conducted in a private clinical lab. By evaluating the efficacy and reliability of rapid molecular-based diagnostics, we hope to provide insight on how to enhance clinical practice, strengthen antimicrobial stewardship efforts, build clinicians’ confidence in rapidly generated results, and accelerate access to appropriate therapies in settings with high rates of antimicrobial resistance. Peru’s healthcare landscape, marked by a high burden of antimicrobial resistance and limited resources, provides a critical setting for evaluating the impact of these diagnostic strategies [5]. Findings provided by this study may lead to implementations of CDSS and diagnostic models elsewhere that can significantly reduce the time to optimal therapy [23].

## 2. Materials and Methods

### 2.1. Study Design and Setting

This study was designed as a prospective, observational, cross-sectional analysis to evaluate the precision of therapeutic recommendations generated by two distinct systems: AOCHMR, which uses molecular results to provide treatment guidance to clinicians, and AOCHFR, which similarly offers guidance but incorporates both phenotypic and molecular results. The study was conducted at a private clinical laboratory in Lima, Peru, from August 2024 to December 2024. A cross-sectional design was chosen to assess the agreement between the two diagnostic approaches within a defined timeframe, enabling a direct comparison under consistent laboratory conditions.

### 2.2. Study Population

Participants in this study were patients diagnosed with bacteremia, identified by at least one positive blood culture for a known pathogenic organism. Selection criteria included adults aged 18 years and older, presenting with clinical signs consistent with bloodstream infection. Patients were excluded if they had invalid phenotypic identification or antibiogram results using MALDI-TOF or VITEK 2.0, or if they lacked preliminary AOCHMR or final AOCHFR recommendations (scenarios where no recommendations were provided). The study aimed to minimize selection bias by including consecutive patients meeting the inclusion criteria during the study period.

### 2.3. Data Acquisition and Description

Blood samples were collected from patients with suspected bacteremia and processed for blood cultures. The initial positive blood culture bottles, corresponding to the earliest positivity alarm, were selected for analysis. The study employed two principal testing methodologies:

Molecular Testing: Positive blood culture samples underwent rapid molecular analysis using the FilmArray Blood Culture Identification (BCID) Panel (BioFire Diagnostics, LLC, Salt Lake City, UT, USA) or Xpert^®^ MRSA/SA Blood Culture (Cepheid LLC, Sunnyvale, CA, USA), selected based on Gram stain results. The assays were conducted according to the manufacturer’s instructions, with specific attention to reagent preparation, sample volume (200 µL), and assay run conditions (temperature and duration).

Phenotypic Testing: Organisms from positive blood cultures were isolated on agar media, including Blood Agar, Chocolate Agar, McConkey Agar, and Sabouraud Agar. Microbial identification was performed using the Matrix-assisted laser desorption ionization-time of flight mass spectrometry (MALDI-TOF MS, Bruker, Billerica, MA, USA) (bioMérieux) system, calibrated daily to ensure accuracy. Antimicrobial susceptibility testing (AST) was conducted using the VITEK 2.0 (bioMérieux SA, Lyon, France) automated system, with the results integrated to generate AOCHFR recommendations.

### 2.4. Study Procedures and Tools/Instruments/Materials/Equipment Molecular Testing Procedures

1. Sample Preparation: A 200 µL aliquot of positive blood culture was prepared for analysis. The sample was mixed with a lysis buffer to release nucleic acids.
2. FilmArray/GeneXpert Assay: The prepared sample was loaded into the FilmArray or GeneXpert cartridge and inserted into the instrument. The system performed automated nucleic acid extraction, amplification, and detection, providing results within approximately 2 h.
3. Pathogen Identification and Resistance Detection: The system identified pathogens and detected antimicrobial resistance genes, generating an AOCHMR with therapeutic recommendations based solely on molecular findings.

#### Phenotypic Testing Procedures

1. Culture and Isolation: Positive blood culture samples were streaked onto agar plates and incubated at 37 °C for 18–24 h. Colonies were examined for morphological characteristics.
2. MALDI-TOF Identification: A single colony was applied to a MALDI-TOF target plate, overlaid with a matrix solution, and analyzed by the mass spectrometer. The system matched the obtained spectra to a reference database for organism identification.
3. VITEK 2.0 AST: Isolated organisms were suspended in saline to a McFarland standard of 0.5 and loaded into the VITEK 2.0 system for AST. The system provided results within 8–12 h, which were used to refine AOCHFR therapeutic recommendations.

### 2.5. Data Preparation

Data from molecular and phenotypic testing were compiled into a centralized database (Supplement S1 in Supplementary Materials). Each patient’s results were anonymized using numeric codes to maintain confidentiality. Data cleaning involved verifying the consistency of test results, checking for missing values, and resolving discrepancies between molecular and phenotypic findings.

### 2.6. Data Analysis

Statistical analyses were conducted using Stata v17 software (StataCorp., College Station, TX, USA). Categorical variables, such as gender and concordance measures, were reported as frequencies and percentages. Continuous variables, including age, number of bottles taken, time to the first alert, AOCHMR time, AOCHFR time, and time difference, were summarized using median and interquartile range (IQR).

**Figure 1:**
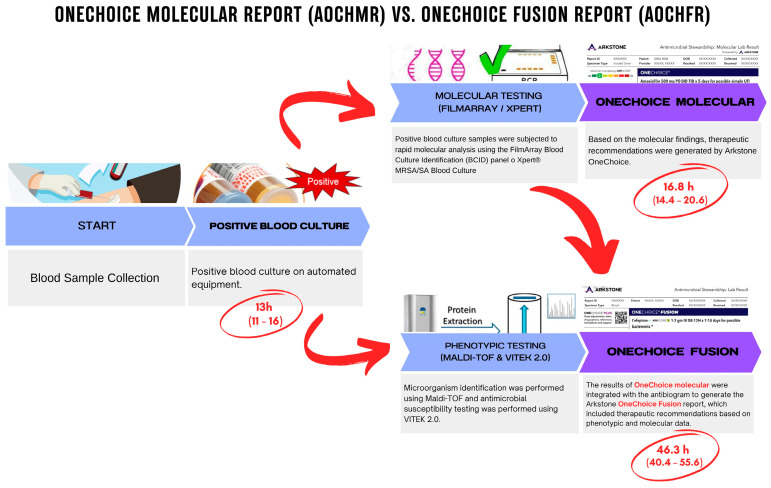
Workflow and time comparison: AOCHMR vs. AOCHFR.

The results generated an AOCHMR (Supplement S2), which included therapeutic recommendations based solely on molecular findings (Figure 1).

Results were integrated to generate AOCHFR (Supplement S3), which included refined therapeutic recommendations based on phenotypic and molecular data (Figure 1). Both AOCHMR and AOCHFR provided primary therapeutic recommendations that were considered optimal and preferred (called OneChoice), and secondary recommendations (called Alternative Treatment Options).

### 2.7. Statistical Techniques

1. Concordance Analysis**: Cohen’s Kappa was used to measure the agreement between the therapeutic recommendations of AOCHMR and AOCHFR. This analysis provided insight into the consistency and reliability of the molecular-only versus combined molecular and phenotypic approaches.
2. Regression Analysis: Poisson regression was employed to analyze factors influencing concordance between AOCHMR and AOCHFR recommendations. This included controlling for potential confounders such as age, gender, number of positive vials, time differences, and specific bacteriological factors. Poisson regression was chosen based on its suitability for modeling count data and the presence of overdispersion in the outcome variable.
3. Time Comparison: A paired non-parametric test (Mann–Whitney U) was conducted to evaluate the time efficiency of AOCHMR versus AOCHFR recommendations. The time difference in hours between the two systems was analyzed to provide insights into the potential clinical advantages of each diagnostic approach.

### 2.8. Ethical Considerations

**Figure 2:**
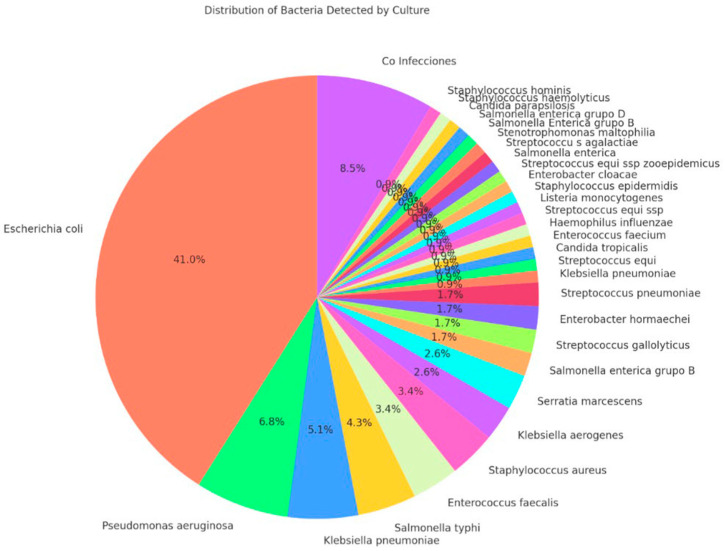
Distribution of detected bacteria.

**Figure 3:**
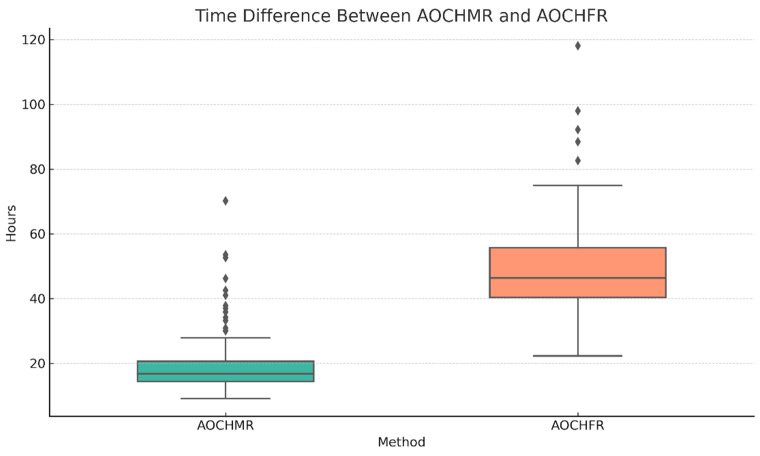
Time difference between AOCHMR and AOCHFR.

**Figure 4:**
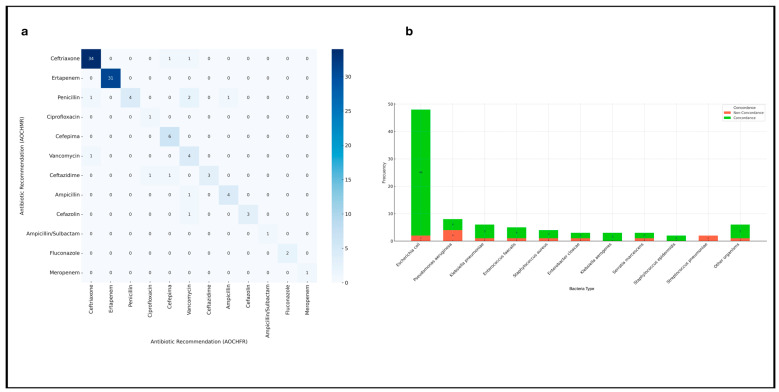
(**a**) Confusion matrix for antibiotic recommendations between AOCHMR and AOCHFR; (**b**) Concordance in therapeutic recommendations between AOCHMR and AOCHFR grouped by bacteria.

The Faculty of Health Sciences Ethics Committee at the Universidad Privada de Tacna approved the study protocol. Given the study’s reliance on secondary analysis of de-identified data, the requirement for patient consent was waived. All patient data were anonymized using numeric codes to ensure confidentiality and compliance with ethical standards. The ethical considerations were guided by principles of respect for persons, beneficence, and justice, ensuring that the research adhered to high ethical standards while minimizing risks to participants. We used Generative AI to make Figures 2–4.

## 3. Results

A total of 117 patients with bacteremia were enrolled in this study to evaluate the concordance between therapeutic recommendations generated by AOCHMR and AOCHFR. The median age of the study population was 67 years, with an interquartile range (IQR) of 45 to 79 years, and there was a slightly higher proportion of males (58.12%) compared to females (41.88%). On average, two blood culture bottles were taken per patient (IQR: 2–4), with a median of two bottles testing positive (IQR: 1–2). The median time to the first alert for bacterial growth was 13 h (IQR: 11–16), as detailed in Table 1, which comprehensively summarizes the study population’s demographic, clinical, and bacteriological characteristics and a time-to-result analysis. This table is critical for understanding the baseline characteristics of the cohort and the efficiency of the diagnostic methods employed.

The AOCHMR and AOCHFR methods utilized MALDI-TOF mass spectrometry for bacterial identification, successfully identifying 117 bacterial species. The concordance between the two methods for species detection was high, at 86.32%. Resistance mechanism detection also showed an 86.32% concordance rate. *Escherichia coli* was the most prevalent bacterial species identified, accounting for 41.0% of cases, followed by *Pseudomonas aeruginosa* (6.8%) and *Klebsiella pneumoniae* (5.1%). These findings are visually represented in Figure 2, illustrating the distribution of bacterial species identified in the study.

### 3.1. Time to Recommendation

The time to generate therapeutic recommendations differed significantly between the two systems. The median time for the AOCHMR was 16.81 h (IQR: 14.38–20.58), whereas the AOCHFR report required a median time of 46.32 h (IQR: 40.41–55.69). The median difference in time to results between the two methods was 28.43 h (IQR: 22.93–34.89), as shown in Figure 3. This substantial difference in reporting time highlights the potential clinical advantage of the AOCHMR system in providing rapid therapeutic guidance.

### 3.2. Concordance of Therapeutic Recommendations

Primary therapeutic recommendations between AOCHMR and AOCHFR showed a high level of agreement, with concordance observed in 80.34% of cases and discordance in 19.66%. However, agreement for alternative therapeutic options was lower, with only 48.71% concordance and 51.29% discordance, as detailed in Table 1. The overall Cohen’s Kappa index for therapeutic recommendations was 0.80, indicating substantial agreement between the two systems. Agreement was particularly strong for commonly recommended antibiotics: Ceftriaxone and ertapenem had the highest concordance, with 34 and 31 matching cases, respectively. Moderate agreement was observed for antibiotics like cefepime (six cases) and vancomycin (four cases). Some discrepancies were noted, particularly with penicillin, which, despite four concordant cases, also showed mismatches when alternatives like ampicillin were recommended instead, or in scenarios where no primary option could be provided at all. These findings are further illustrated in Figure 4a, which provides a detailed side-by-side comparison of antibiotic recommendations between the two systems.

Poisson regression analysis was conducted to identify variables associated with the prevalence of concordance between the therapeutic recommendations of AOCHMR and AOCHFR. Two variables were significantly associated with concordance in the crude analysis: the presence of three positive blood culture bottles (crude Prevalence Ratio [cPR] = 1.20; 95% Confidence Interval [CI]: 1.04–1.37; *p* = 0.009) and the isolation of *Pseudomonas aeruginosa* (cPR = 0.50; 95% CI: 0.249–1.002; *p* = 0.05). However, in the adjusted analysis, only the isolation of *Streptococcus* remained statistically significant, with its presence associated with a lower prevalence of concordance (adjusted Prevalence Ratio [aPR] = 0.40; 95% CI: 0.16–0.98; *p* = 0.04). These results are summarized in Table 2, which presents the crude and adjusted prevalence ratios for concordance with primary therapeutic recommendations.

Our results demonstrate that the AOCHMR system delivers rapid, reliable therapeutic recommendations with substantial concordance to the AOCHFR system, especially for primary treatment options. These findings highlight AOCHMR’s potential to improve clinical decision-making by providing timely, accurate guidance for therapy selection.

## 4. Discussion

This study presents a comparative evaluation of two CDSS approaches (AOCHMR and AOCHFR) based on AI and ML, aimed at recommending antimicrobial treatments in patients with bacteremia, using either molecular diagnostic results alone or combined with phenotypic susceptibility data. Our findings reveal a high level of concordance between the two systems for initial therapeutic recommendations, with an agreement rate of 80.34%. The discrepancies may be due to the inability of some molecular systems to detect phenotypic resistance, which could lead to differences in antibiotic therapy if not complemented with phenotypic susceptibility data [24]. Additionally, there was a consistency of 86.32% in detecting bacterial species and resistance genes. This supports previous studies highlighting that complementing molecular testing with conventional methods improves diagnostic accuracy [25]. A significant advantage of the AOCHMR system was its speed, delivering results approximately 29 h faster than the AOCHFR system, underscoring the potential of rapid molecular diagnostics in guiding antimicrobial therapy decisions in bacteremia.

The high concordance observed between AOCHMR and AOCHFR suggests that rapid molecular testing, coupled with a robust Clinical Decision Support System (CDSS), can be a reliable tool in the early management of bacteremia. This is particularly critical in conditions like sepsis and bacteremia, where delayed antimicrobial administration is associated with increased mortality, as noted by Bonine et al. [6]. AOCHMR provided clinicians with actionable therapeutic recommendations within approximately 16.81 h post-blood culture collection, a crucial timeframe for such time-sensitive conditions. In contrast, the AOCHFR system took nearly 46 h. This rapid turnaround offers a significant opportunity to improve outcomes for bacteremia patients, especially those who are critically ill. Importantly, our findings demonstrate that relying solely on molecular methods can provide correct recommendations in 80% of bacteremia cases, 29 h earlier than conventional methods, and this accuracy can reach up to 95% depending on the detected bacteria and resistance genes.

The analysis of time differences between the two testing methods further emphasizes the clinical relevance of molecular diagnostics. Our findings align with those reported by Holma et al. and Lau et al. [10,26], showcasing the capability of molecular methods to swiftly identify pathogens and resistance genes, thereby reducing the time to therapeutic recommendations. In this study, we demonstrated reducing the time to therapeutic recommendations by approximately 29 h. Rapid molecular assays and mass spectrometry for identifying bacterial species and susceptibility in blood cultures have been associated with statistically significant improvements in initiating appropriate antibiotic therapy [27,28], reduced rates of recurrent infections [29], decreased mortality, shorter hospital stays, and lower hospital costs [30,31]. In regions like Peru, where antimicrobial resistance (AMR) is highly prevalent, the timely initiation of appropriate therapy is crucial for reducing morbidity and mortality [5].

Despite the high level of agreement, our findings revealed a discordance rate of 19.66% in primary and 51.29% in alternative antibiotic recommendations between the molecular-based and molecular–phenotypic approaches. This highlights the need for integrated approaches using both genotypic and phenotypic results for optimal antimicrobial recommendations, particularly in complex resistance patterns, as described by Tamma et al. and Claeys et al. [32,33]. The gap in concordance is partly due to the inability of molecular methods alone to capture all phenotypic resistance patterns, as noted by Holma et al. and Banerjee et al. [10,34]. In this context, AOCHFR provides an additional layer using phenotypic data, which can further refine antibiotic recommendations.

Our Poisson regression analysis indicated that the isolation of Pseudomonas aeruginosa significantly influences the agreement between both systems, underscoring the variability in test sensitivity and specificity among different bacterial species. Pseudomonas aeruginosa may present a more complex resistance profile, as described by Qin et al. and Giovagnorio et al. [23,35], reinforcing the need for a personalized approach to CDSS implementation that accounts for prevalent pathogens and associated resistance in specific contexts [36]. However, the variability in resistance did not significantly affect the consistency between both systems, demonstrating the robustness of the CDSS.

The exceptionally high concordance observed in therapeutic recommendations for *Escherichia coli* (Figure 4b), which was the most frequently isolated pathogen, is noteworthy. This suggests that the AOCHMR is highly reliable in guiding treatment for common bacteremia cases, providing appropriate antimicrobial guidance much faster than traditional methods. This accelerated diagnostic pathway is crucial for improved antimicrobial stewardship, allowing clinicians to quickly administer targeted therapy and reduce reliance on broad-spectrum antibiotics, which can contribute to resistance and increase risks of adverse reactions. The shift away from empirical treatment protocols marks a new paradigm in bacteremia management. Studies show that AI, ML, and CDSS enhance antimicrobial stewardship, while rapid diagnostic tests for bloodstream infections highlight the critical importance of timely, targeted therapy to improve patient outcomes [19,37,38].

A Cohen’s kappa index of 0.80 for therapeutic recommendations aligns with findings from other diagnostic concordance studies, indicating a high level of agreement between the two systems. This is especially significant because the system incorporating both phenotypic and molecular data is considered the gold standard, making the molecular-based system’s ability to deliver similar recommendations in a shorter time particularly noteworthy.

AOCHMR, utilizing molecular data with AI/ML-powered CDSS, could represent a paradigm shift in the management of bacteremia. The ability to obtain reliable therapeutic recommendations in less than 20 h represents a significant advancement compared to the nearly 49 h required by conventional phenotypic methods. This rapid decision-making and a high degree of consistency hold important implications for clinicians. This method could mitigate delays in appropriate therapy, particularly in settings with high AMR rates, and improve patient outcomes. Moreover, the system can contribute to better antimicrobial stewardship practices by reducing the dependence on empirical therapies, a significant clinical dilemma when managing critically ill patients, as described by De Angelis et al. and further supported by Blechman and Wright [19,39].

Despite the robust nature of our study, there are limitations to consider. First, this was a single-center study conducted in a private laboratory, which may limit the generalizability of our findings to other settings. Additionally, the relatively small sample size and the exclusion of cases without a treatment recommendation from either algorithm may introduce some selection bias. Second, we did not directly analyze clinical outcomes; therefore, further research is needed to explore the system’s impact on mortality, morbidity, and length of hospital stay. Future studies should investigate the real-world clinical impact of implementing AOCHMR, including its effect on these key outcomes. Although current research indicates that barriers to acceptance of these models persist among healthcare professionals—as these are still evolving tools [40]—it is also critical to assess the cost-effectiveness of the rapid molecular CDSS and to evaluate how specific antimicrobial resistance patterns may influence the system’s performance. Moreover, conducting prospective studies of real-time clinical outcomes in controlled trials will provide valuable additional information.

It is also important to note that, in real-world clinical practice, therapeutic decisions are not based solely on diagnostic test results, whether molecular or phenotypic. Clinical judgment integrates multiple variables, including comorbidities, severity of illness, prior antimicrobial treatments, site of infection, and local epidemiological patterns. The purpose of this study was to evaluate, under controlled conditions, the diagnostic value and therapeutic accuracy of rapid molecular data for guiding antimicrobial treatment. Our findings should not be interpreted as a replacement for clinical judgment, but rather as a complementary tool that may contribute to early therapeutic decision-making.

Finally, integrating additional clinical factors beyond those currently considered by the AI/ML CDSS into the treatment decision-making algorithm may offer a more nuanced approach to antimicrobial recommendations, something that is already being explored.

Our findings demonstrate that the AOCHMR system provides rapid and reliable therapeutic recommendations with substantial concordance with the AOCHFR system, particularly for primary therapeutic options. The results underscore the potential of AOCHMR to enhance clinical decision-making by delivering timely and accurate treatment guidance.

## 5. Conclusions

Our findings demonstrate that the AOCHMR, which utilizes AI/ML-CDSS based solely on molecular data, provides rapid and accurate therapeutic recommendations for bacteremia, addressing the critical need for timely intervention in high antimicrobial resistance settings. This study makes an important contribution to the field of infectious disease by demonstrating how AI/ML-driven systems and molecular methods can streamline decision-making in infectious disease management, ultimately reducing the time to effective treatment and improving patient outcomes. Integrating these technologies into clinical practice has the potential to revolutionize antimicrobial stewardship by enabling more precise and timely therapeutic strategies, ultimately shaping healthcare policies to prioritize the adoption of advanced diagnostic tools.

Future research should focus on expanding the capabilities of AI/ML-powered systems by incorporating additional data types, such as patient demographics and environmental factors, to enhance predictive accuracy. Furthermore, longitudinal studies assessing the real-world impact of these systems on clinical outcomes and resistance patterns across diverse healthcare settings are essential. Investigating the cost-effectiveness and scalability of implementing AI/ML-driven diagnostics in resource-limited environments will also provide valuable insights. Building on these findings, future studies can further refine CDSS applications in healthcare, enhancing their effectiveness and ensuring broader adoption in the management of complex infectious diseases.

## Figures and Tables

**Table 1:** Demographics, clinical and bacteriological findings, and time-to-result analysis in patients with bacteremia by concordance of primary therapeutic recommendations between AOCHMR and AOCHFR.

Variable	Total (*n* = 117)	Non-Concordance (*n* = 23)	Concordance (*n* = 94)	*p*-Value
Demographics and Clinical Characteristics				
- Age (years) 1	67 (45–79)	69 (45–79)	65.5 (46–80)	0.898 a
- Male gender (%)	68 (58.12)	13 (56.52)	55 (58.51)	0.862 b
- Blood culture bottles collected per patient 1	2 (2–4)	2 (2–4)	2 (2–4)	0.999 a
- Positive blood culture bottles per patient 1	2 (1–2)	2 (1–2)	2 (1–2)	0.822 a
- Time to first alert (hours) 1	13 (11–16)	13 (12–16)	13 (11–16)	0.439 a
Bacteriological and Molecular Results				-
- Bacteria detected by molecular method (%)	117 (100.0)	23 (100.0)	94 (100.0)	
- Bacteria detected by conventional culture (%)	117 (100.0)	23 (100.0)	94 (100.0)	
- Concordance in bacterial identification (%)	101 (86.32)	16 (69.56)	85 (90.42)	0.027 b
- Concordance in bacterial resistance identification (%)	101 (86.32)	16 (69.56)	85 (90.42)	0.011 b
Time comparison				
- AOCHMP time (hours) 1	16.81 (14.38–20.58)	18.02 (15.98–20.33)	16.62 (14.17–20.68)	0.434 a
- AOCHFR time (hours) 1	46.32 (40.41–55.69)	47.83 (42.92–66.95)	45.84 (39.85–54.25)	0.111 a
- Time difference (hours) 1	28.43 (22.93–34.89)	29.57 (23.85–43.68)	28.09 (22.61–34.42)	0.246 a
Concordance of Therapeutic Recommendations				
- Primary recommendation concordance (%)	94 (80.34)	-	-	-
- Alternative recommendation concordance (%)	57 (48.71)	4 (17.39)	53 (56.38)	0.002 b

median and interquartile range, a = U-Mann–Whitney test, b = chi-squared test. Bacterial identification and Resistance mechanism Detection.

**Table 2:** Poisson regression analysis evaluates crude and adjusted prevalence ratios of concordance with primary therapeutic recommendations between AOCHMR and AOCHFR in bacteremias.

Variable	cPR (95% CI)	*p*-Value	aPR (95% CI)	*p*-Value
Age	0.999 (0.996–1.003)	0.88	-	
Male gender	1.016 (0.845–1.221)	0.86	-	
Blood culture bottles collected per patient		0.82	-	
- Two	Reference			
- Four	0.977 (0.802–1.190)			
Positive blood culture bottles per patient			-	
- Two	0.918 (0.758–1.113)	0.38		
- Three	1.200 (1.047–1.374)	<0.01	0.954 (0.858–1.060)	0.38
- Four	1.066 (0.815–1.395)	0.63		
Time to result of molecular (hours)	1.001 (0.993–1.008)	0.76	-	
Time to result of phenotype	0.997 (0.991–1.002)	0.32	-	
Bacteria detected by conventional culture				
- *Escherichia coli*	0.958 (0.903–1.016)	0.15		
- *Salmonella* spp.	0.800 (0.586–1.092)	0.16		
- *Klebsiella* spp.	0.888 (0.704–1.120)	0.32		
- *Pseudomonas aeruginosa*	0.500 (0.249–1.002)	0.05	0.545 (0.272–1.091)	0.08
- *Streptococcus* spp.	0.375 (0.152–0.920)	0.03	0.408 (0.169–0.988)	0.04
- Polymicrobial	0.750 (0.501–1.120)	0.16		

cPR: crude Prevalence Ratio; 95% CI: 95 percent confidence interval.

## Data Availability

The data analyzed in this manuscript, as well as its definitions, can be seen in Supplement S1 in Supplementary Materials.

## Supplementary Materials

The following supporting information can be downloaded at https://www.mdpi.com/article/10.3390/life15060864/s1, Supplement S1: Data base in Excel File, Supplement S2: OneChoice Molecular In PDF File, Supplement S3: OneChoice Fusion In PDF File.

## Abbreviations

The following abbreviations are used in this manuscript:

AI	Artificial intelligence
ML	Machine learning
CDSS	Clinical decision support system
BSIs	Bloodstream infections
AMR	Antimicrobial resistance
AOCHMR	Arkstone’s OneChoice Molecular report
AOCHFR	Arkstone’s OneChoice Fusion report
