# Supplementary material for: AI-Based Treatment Recommendations Enhance Speed and Accuracy in Bacteremia Management: A Comparative Study of Molecular and Phenotypic Data
Source: Life (Basel). 2025 May 27;15(6):864. doi: 10.3390/life15060864 (PMC12194749; doi:10.3390/life15060864)
Supplement: Supplementary file 1 [file life-15-00864-s001.zip › Supplement S2.pdf]

Administración antimicrobiana: Resultado molecular

Desarrollado por ARKSTONE

**ID de informe**	93X9	**Paciente**	XXXX	**FDN**	10/11/19X1	**Recogido**	30/8/2024
**Tipo De Espécimen**	Blood	**Instalaciones**	Laboratorios Roe	**Resultado**	30/8/2024	**Recibió**	30/8/2024

ARKSCORE™ de complejidad de infección

LO 2 HI

Organismos detectados

Patógenos comunes en negrita

- **Escherichia coli**

Resistencia detectada

Extended-Spectrum Beta-Lactamase

ARKSCORE™ de resistencia

LO 3 HI

No se reportaron alergias

Información de la droga

Ertapenem

Ajustes de dosis ☑ Renal ☐ Hepático

Efectos secundarios DRESS syndrome

Interacciones Valproic acid

ARKSCORE™ de reacciones adversas

LO 3 HI

ONECHOICE® PLUS

Ajustes de dosis, opciones de drogas, referencias, traducciones y apoyo

ONECHOICE®

Ertapenem 1 g IV cada 24 horas durante 7 a 14 días por posible bacteriemia

Opciones de tratamiento alternativas con Arkscore de reacciones adversas™

- **Gentamicina°** ARKSCORE 5 5-7 mg/kg/dosis IV/IM cada 24 horas (ajustado de pico y valle) durante 7-14 días
- **Meropenem°** ARKSCORE 3 1-2 g IV cada 8 horas durante 7-14 días
- **Cipro°** ARKSCORE 5 400 mg IV cada 12 horas durante 7 a 14 días

° Consulte información de dosificación adicional en OneChoice Plus. La eficacia de Gentamicina contra E. coli es incierta.

¿Por qué es esta la OneChoice?

E. coli puede ser patógena cuando se encuentra en muestras de sangre. Se detectaron genes de resistencia que pueden limitar las opciones de tratamiento disponibles. El tratamiento recomendado es óptimo ya que se dirige a todos los microbios afectados. ‡

¿Cuándo se debe tratar esto?

Las bacterias en la sangre siempre deben tratarse cuando la contaminación no sea motivo de preocupación. Se debe determinar la fuente de la bacteriemia para garantizar que la infección sea tratada adecuadamente, ya que es posible que sea necesario adaptar los antibióticos específicamente a la fuente de la infección. Para muchos microbios, no es posible la de-escalación a antibióticos orales. La duración del tratamiento depende de la fuente de infección, pero generalmente es de 7 a 14 días como mínimo y cuando la infección es complicada puede extenderse por varias semanas. ‡

¿Hay alguna consideracion especial?

Como la resistencia a ESBL está en la lista de amenazas de los CDC, se puede indicar seguimiento y monitoreo, si es posible. La BLEE puede asociarse con otros genes de resistencia. Por lo tanto, los antibióticos deben utilizarse con precaución ya que es posible que el medicamento falle. Los hemocultivos suelen ser positivos en presencia de infección. Sin embargo, pueden producirse falsos positivos debido a la contaminación. La resistencia detectada puede afectar sólo a ciertos microbios y, en algunos casos, a ninguno en absoluto. ‡

Precauciones para el control de infecciones: ☑ Estándar ☑ Contacto

* Posología y duración del tratamiento en función de paciente adulto, sin antecedentes médicos, con IMC, función renal y hepática normales, y mínimo tiempo requerido para tratar infecciones simples. El tratamiento está dirigido a los patógenos comunes mencionados anteriormente y la resistencia a los antibióticos más comúnmente asociada con base en los genes detectados. Es posible que se necesite un estudio microbiológico adicional y una modificación del tratamiento.

‡ Solo con fines educativos. Esto no es un diagnóstico. Se requiere correlación clínica y juicio médico al tomar decisiones de diagnóstico o tratamiento. Recomendaciones basadas únicamente en los datos recibidos. No se ha examinado al paciente ni se ha revisado su historial médico.

Copyright 2025 Arkstone Medical Solutions. OneChoice, MedsMatrix y ArkScore se basan en métodos y algoritmos pendientes de patente. Aprende más: arkstone.ai/report
