# Supplementary material for: AI-Based Treatment Recommendations Enhance Speed and Accuracy in Bacteremia Management: A Comparative Study of Molecular and Phenotypic Data
Source: Life (Basel). 2025 May 27;15(6):864. doi: 10.3390/life15060864 (PMC12194749; doi:10.3390/life15060864)
Supplement: Supplementary file 1 [file life-15-00864-s001.zip › Supplement S3.pdf]

ROE Laboratorio

Administración antimicrobiana: Resultado de laboratorio

Desarrollado por ARKSTONE

ID de informe	94X8	Paciente	XXXX	FDN	10/11/19X1	Recogido	30/8/2024
Tipo De Espécimen	Blood	Instalaciones	Laboratorios Roe	Resultado	31/8/2024	Recibió	30/8/2024

ONECHOICE® PLUS
Ajustes de dosis, opciones de drogas, referencias, traducciones y apoyo

ARKSCORE™ de complejidad de infección

LO 2 HI

Organismos detectados

Patógenos comunes en negrita

- **Escherichia coli**

Resistencia detectada

Extended-Spectrum Beta-Lactamase

ARKSCORE™ de resistencia

LO 3 HI

No se reportaron alergias

ONECHOICE® FUSION

Ertapenem ARKSCORE 3 1 g IV cada 24 horas durante 7 a 14 días por posible bacteriemia *

Opciones de tratamiento alternativas with Adverse Reaction ArkScore™

- **Amikacina** ARKSCORE 5 , 15 a 30 mg/kg IV cada 24 horas (luego el intervalo de dosificación se basa en la evaluación farmacocinética) durante 7 a 14 días (no suele usarse como monoterapia)
- **Cef/Sul°** ARKSCORE 3 2 a 4 g/día IV/IM en dosis divididas equitativamente cada 12 horas durante 7 a 14 días (según datos limitados, es posible que falle el medicamento)

° La eficacia de Cef/Sul contra E. coli es incierta.

¿Por qué es esta la OneChoice?

La E. coli puede ser patógena cuando se encuentra en muestras de sangre. Se detectaron genes de resistencia que pueden limitar las opciones de tratamiento disponibles. El tratamiento recomendado es óptimo, ya que ataca a todos los microbios en cuestión. La amikacina debe utilizarse con precaución, ya que se ha descubierto que otros aminoglucósidos son resistentes y, por lo tanto, es posible que el fármaco no funcione. ‡

¿Cuándo se debe tratar esto?

Las bacterias en la sangre siempre deben tratarse cuando la contaminación no sea motivo de preocupación. Se debe determinar la fuente de la bacteriemia para garantizar que la infección sea tratada adecuadamente, ya que es posible que sea necesario adaptar los antibióticos específicamente a la fuente de la infección. Para muchos microbios, no es posible la de-escalación a antibióticos orales. La duración del tratamiento depende de la fuente de infección, pero generalmente es de 7 a 14 días como mínimo y cuando la infección es complicada puede extenderse por varias semanas. ‡

¿Hay alguna consideracion especial?

Como la resistencia a ESBL está en la lista de amenazas de los CDC, se puede indicar seguimiento y monitoreo, si es posible. La BLEE puede asociarse con otros genes de resistencia. Por lo tanto, los antibióticos deben utilizarse con precaución ya que es posible que el medicamento falle. Los hemocultivos suelen ser positivos en presencia de infección. Sin embargo, pueden producirse falsos positivos debido a la contaminación. La resistencia detectada puede afectar sólo a ciertos microbios y, en algunos casos, a ninguno en absoluto. ‡

Control de infección: ☑ Estándar ☑ Contacto

* Posología y duración del tratamiento en función de paciente adulto, sin antecedentes médicos, con IMC, función renal y hepática normales, y mínimo tiempo requerido para tratar infecciones simples. El tratamiento está dirigido a los patógenos comunes mencionados anteriormente y la resistencia a los antibióticos más comúnmente asociada con base en los genes detectados. Es posible que se necesite un estudio microbiológico adicional y una modificación del tratamiento.

‡ Solo con fines educativos. Esto no es un diagnóstico. Se requiere correlación clínica y juicio médico al tomar decisiones de diagnóstico o tratamiento. Recomendaciones basadas únicamente en los datos recibidos. No se ha examinado al paciente ni se ha revisado su historial médico.

Copyright 2025 Arkstone Medical Solutions. OneChoice, MedsMatrix y ArkScore se basan en métodos y algoritmos pendientes de patente. Aprende más: arkstone.ai/report

ROE Laboratorio

Administración antimicrobiana: Resultado de laboratorio

Desarrollado por ARKSTONE

**ID de informe**	94X8	**Paciente**	XXXX	**FDN**	10/11/19X1	**Recogido**	30/8/2024
**Tipo De Espécimen**	Blood	**Instalaciones**	Laboratorios Roe	**Resultado**	31/8/2024	**Recibió**	30/8/2024

MEDSMATRIX™	1	2	3	4	5	6	7	8	9	10	11	12
Escherichia coli	R (genes)	R (genes)	R (genes)	R (genes)	S (genes)	S	S	R	?	S	R	R

S Susceptible
I Indeterminado
Actividad conocida según las directrices actuales
Variable activity based on current guidelines
R Resistente
Genes de resistencia detectados
Impedido por alergia a medicamentos reportada
— No documented activity

1 Ceftriaxona

Ajustes de dosis ☐ Renal ☑ Hepático
Efectos secundarios Pseudocholelithiasis
Interacciones Prevacid
ARKSCORE™ de reacciones adversas
LO 2 HI

2 Ampicilina/Sulbactam

Ajustes de dosis ☑ Renal ☐ Hepático
Efectos secundarios Eosinophilia
Interacciones Allopurinol
ARKSCORE™ de reacciones adversas
LO 3 HI

3 Ceftazidima

Ajustes de dosis ☑ Renal ☐ Hepático
Efectos secundarios Sunburn
Interacciones None
ARKSCORE™ de reacciones adversas
LO 3 HI

4 Cefepima

Ajustes de dosis ☑ Renal ☐ Hepático
Efectos secundarios Positive Coombs
Interacciones None
ARKSCORE™ de reacciones adversas
LO 3 HI

5 Piperacilina/Tazobactam

Ajustes de dosis ☑ Renal ☐ Hepático
Efectos secundarios Thrombocytopenia
Interacciones Methotrexate
ARKSCORE™ de reacciones adversas
LO 4 HI

6 Ertapenem

Ajustes de dosis ☑ Renal ☐ Hepático
Efectos secundarios DRESS syndrome
Interacciones Valproic acid
ARKSCORE™ de reacciones adversas
LO 3 HI

7 Meropenem

Ajustes de dosis ☑ Renal ☐ Hepático
Efectos secundarios Diarrhea
Interacciones Valproic acid
ARKSCORE™ de reacciones adversas
LO 3 HI

8 Gentamicina

Ajustes de dosis ☑ Renal ☐ Hepático
Efectos secundarios Renal/Cochlear/Vestibular toxicity
Interacciones Lasix/ NSAIDS
ARKSCORE™ de reacciones adversas
LO 5 HI

9 Tobramicina

Ajustes de dosis ☑ Renal ☐ Hepático
Efectos secundarios Renal/Cochlear/Vestibular toxicity
Interacciones Lasix/ NSAIDS
ARKSCORE™ de reacciones adversas
LO 4 HI

10 Amikacina

Ajustes de dosis ☑ Renal ☐ Hepático
Efectos secundarios Renal/Cochlear/Vestibular toxicity
Interacciones Lasix/NSAIDS
ARKSCORE™ de reacciones adversas
LO 5 HI

11 Levofloxacina

Ajustes de dosis ☑ Renal ☐ Hepático
Efectos secundarios Tendinitis
Interacciones Cations
ARKSCORE™ de reacciones adversas
LO 5 HI

12 Ciprofloxacina

Ajustes de dosis ☑ Renal ☐ Hepático
Efectos secundarios Tendinitis
Interacciones Cations
ARKSCORE™ de reacciones adversas
LO 5 HI

Escanee o haga clic para visitar **ONECHOICE® PLUS** y ver 1 tratamiento adicional opción en línea

* Posología y duración del tratamiento en función de paciente adulto, sin antecedentes médicos, con IMC, función renal y hepática normales, y mínimo tiempo requerido para tratar infecciones simples. El tratamiento está dirigido a los patógenos comunes mencionados anteriormente y la resistencia a los antibióticos más comúnmente asociada con base en los genes detectados. Es posible que se necesite un estudio microbiológico adicional y una modificación del tratamiento.

‡ Solo con fines educativos. Esto no es un diagnóstico. Se requiere correlación clínica y juicio médico al tomar decisiones de diagnóstico o tratamiento. Recomendaciones basadas únicamente en los datos recibidos. No se ha examinado al paciente ni se ha revisado su historial médico.

Copyright 2025 Arkstone Medical Solutions. OneChoice, MedsMatrix y ArkScore se basan en métodos y algoritmos pendientes de patente. Aprende más: arkstone.ai/report
